# Comparison of distally based sural artery and supramalleolar flap for coverage of dorsum of foot and ankle defects; a cross-sectional study of 53 patients

**DOI:** 10.1016/j.amsu.2021.103109

**Published:** 2021-12-04

**Authors:** Pervaiz Mehmood Hashmi, Abeer Musaddiq, Alizah Hashmi, Marij Zahid

**Affiliations:** Aga Khan University Hospital, Karachi, Pakistan

**Keywords:** Soft tissue injuries, Ankle joint, Surgical flaps, Fasciocutaneous flaps, Survival rate

## Abstract

**Background:**

Soft tissue defects over the foot and ankle region are most challenging in reconstructive surgery. Sural artery and supramalleolar flaps have been commonly used for the reconstruction of non-weight-bearing surfaces of the foot. This article aimed to evaluate the long-term outcome comparisons between a sural artery and Supramalleolar flap in the reconstruction of extensive defects of foot and ankle only.

**Methods:**

Between 1996 and 2020, a retrospective analysis of 53 fasciocutaneous flaps (27 sural and 26 Supramalleolar) used for reconstruction of soft tissue defects of foot and ankle were reviewed in this study. The parameters included were demographics data, causes, site and size of the defect, flap size, hospital stay, complications, and outcomes in a pre-structured proforma. The clinical outcome was assessed by a Self-Designed Tool based on flap survival, coverage of defect, weight-bearing status, functional activities of daily living, and cosmetic appearance. Data were analyzed through SPSS version 25.

**Results:**

Among 53 flaps, the major cause of the defect was Trauma (60.4%). The maximum flap size harvested was 25*10 for sural and 20*8 cm for supramalleolar. Complications were seen in 8 (15%) cases in both flaps. Flap tip necrosis and venous congestion were seen in 4 cases. 2 each in Supramalleolar whereas 1 partial necrosis, 1 venous congestion, and 2 infections were seen in the sural artery flap. The flap survival rate in both flaps was 96.2%. Based on the self-designed Tool, flaps were graded Excellent in 43, Good in 8, and Fair in 2 cases. There was no case of Poor in both flaps.

**Conclusion:**

Compared with the sural artery flap, the lateral supramalleolar flap demonstrated higher rates of functional outcomes although flap tip necrosis was higher in Supramalleolar.

## Highlights

• The comparative long-term clinical and functional outcomes of Sural and Supramalleolar flap is based on defect coverage, cosmetic appearance of surgical limb and activity of daily living.
• Compared with the sural artery flap, the lateral Supramalleolar flap is more reliable and exhibit higher rates of functional outcomes.
• Outcomes of Supramalleolar Flap was better than Sural artery flap as it was much more vascular and robust.

## 1 Introduction

The distal lower extremity has a special significance in human function and form. Due to the limited availability and mobility of soft tissues around the foot and ankle, this region of the body is highly susceptible to injury [1]. Soft tissue defects over the foot and ankle commonly occur as a result of trauma, blast injuries, and infection that may involve the bone, tendon, and neurovascular structures [2]. Other causes include machine/industrial injuries, resection of tumors, and neuropathic ulcers [3]**.** Road traffic accidents, gunshots, and bomb blast injuries frequently lead to open fractures with skin loss. Similarly, chronic infections, burns, tumor excision, and skin necrosis around the foot and ankle require coverage with pliable soft tissue for a better range of motion and reconstruction of tendons, nerves, and vessels. These cases are commonly seen in clinical practices that pose a substantial challenge to reconstructive surgeons because of limited local soft tissue availability and underlying infections [4,5] The recommended management of such complex injuries with underlying infection is staged; initial debridement and coverage of defects with local or free flap in the first stage followed by reconstruction of vital structures such as bone, joints, nerves, and tendons. Staged reconstruction provides a better outcome for the restoration of optimal function.

Reconstruction of soft tissue defects of foot and ankle depends on the location, size of the defect, depth of the wound, underlying infection, and associated injuries like bony, ligamentous, and tendon injury/loss. The survival of flaps depends on the surgical techniques and selection of flaps**.** The survival and thickness of the flaps are of chief importance for consideration of postoperative functional recovery and shoe wearing. A certain number of local and local regional flaps including the use of local pedicle flaps, skin-muscle flaps [6], island flaps, fasciocutaneous flaps [7], and autologous free flaps from various parts of the body [[8], [9], [10], [11]]**.** Each method of reconstruction confesses distinct advantages and disadvantages over each other when utilized in distal lower extremity reconstruction**.** Free skin grafts are often unsuitable because they tend to contract and have poor resistance to pressure. Local flaps would be preferable but not suitable due to the small availability of skin, the limited arc of rotation, and reliability [12]. However, fasciocutaneous axial pattern neurocutaneous flaps can still be used. The neurocutaneous sural artery flap is based on the last perforator of the peroneal artery and the supramalleolar flap based on the perforator of a peroneal artery or anterior lateral malleolar artery are ideal flaps to cover the defects of the dorsum of foot, anterior aspect of ankle joint and perimalleolar area. The Distally based sural artery flap was first reported by Hasegawa in 1994 [13]. The sural flap is an island flap based on a sural neurovascular bundle that provides robust axial blood perfusion to flap with significantly greater surface area and ease of transposition [14]. The flap has been designed with reverse flow through the anastomosis between the median superficial sural artery [15,16] and the lowermost perforator of the peroneal artery. It does not sacrifice any major vessel and can be performed without a Doppler flowmeter. The cutaneous artery running along the sural nerve known as the superficial sural artery has a great variation in its location and the origin of the vessel. The lateral supramalleolar flap was first described by Masquelet in 1988 [17] based on the last perforator of the dorsal peroneal artery after it emerges from the interosseous membrane. The cutaneous branch of this perforator supplies the skin over the anterolateral aspect of the lower one-third of the leg. This flap has proved to be a reliable fasciocutaneous flap for the locoregional coverage of the dorsum of the foot, ankle, perimalleolar region, and plantar aspect of the foot without compromising the major blood vessels of the foot including the peroneal artery [18]. However, it is not a suitable option for the weight-bearing region of the heel [19] as the flap is insensate and relatively thin. The flap is most commonly employed as a distally based pedicle island flap.

The introduction of lateral supramalleolar flap and distally based sural artery flaps provides reliable and effective methods to cover the skin defects of the foot and ankle. Due to the reliability of the vascular supply of fasciocutaneous flaps, these flaps have received wide popularity in the reconstructive field, becoming a workhorse for resurfacing the soft-tissue defects in the lower extremities in both anterograde and retrograde way [20] or even in a free approach [21]. In this article, we intend to present our experience comparing a series of the two types of flaps in terms of viability, coverage of defect, cosmetic appearance, and functions of foot and ankle based on a self-designed Tool.

## 2 Methodology

Data of all those patients were collected who underwent reconstruction of soft tissue defects of foot and ankle with Supramalleolar and sural artery flaps between 1996 and 2020. Only defects of foot and ankle around dorsum and perimalleolar areas were included. The defects reconstructed with other local flaps and free flaps were excluded from this review. The data of these patients were collected from the Hospital inpatient management system and review of medical records through a structured proforma that included demographic variables, cause of soft tissue defects (secondary to open fractures, following debridement of chronically infected wounds, tumor excision, and defects following the release of scar contractures), side of defects, size and type of flap (supramalleolar or sural artery), complications and long-term outcome of the flap. All these cases were done by a single surgeon who has experience in microvascular surgery for 20 years. Follow-up records of patients were taken from medical records including immediate complications like venous congestion, hematoma formation, partial or complete necrosis, and wound dehiscence.

The functional outcome for both flaps was assessed retrospectively by a Self-Designed Tool on each patient based on flap survival and coverage of defect, cosmetic acceptance, weight-bearing status, and activity of daily living (ADL) of these patients. Each parameter was divided into four grades: Excellent, good, fair, and poor and each grade was given a specific score of 5, 4, 3, and 2 points respectively. A cumulative score grading system was then assigned and interpreted as excellent for scores 17–20, good for scores 13–16, fair for score 9–12, and poor with score 8 or below.

Categorical variables like gender and mechanism of injury were recorded as frequency and percentages. Discrete and continuous variables like age and flap size etc. were recorded as means and standard deviations. The data were divided into two groups, group 1 consisted of sural artery flaps, and group 2 consisted of the Supramalleolar flap same for foot and ankle defects. The data were checked for normality and the mean differences in the two groups were analyzed independent *t*-test for continuous variables where normality was assumed and Man-Whitney *U* test where normality was not assumed. The Chi-square test was used to analyze categorical variables. Data were recorded and analyzed using Statistical Package for Social Sciences (SPSS) version 25.0. A *p*-value of 0.05 or below was considered significant with a 95% confidence interval. The work has been reported in line with the STROCSS criteria [34] and registered in ClinicalTrial.gov with the Unique Identifying Number (UIN) NCT05027542 [35].

## 3 Dissecting techniques

**Lateral Supramalleolar Flap:** The patient is placed supine with a sandbag under the buttock and a pneumatic tourniquet on the thigh. The skin island is planned according to the defect. The distal limit should include the point of emergence of the perforating branch of the peroneal artery, four finger breadths above the lateral malleolus. The proximal limit reaches the mid-leg. The anterior limit is the tendon of the tibialis anterior muscle and the posterior limit should not cross the posterior border of the fibula. Then an incision is drawn anterior to the lateral malleolus and reaches the depression of the sinus tarsi on the lateral aspect of the hindfoot. First, the anterior margin of the flap is elevated, isolating the pedicle lying on the tibiofibular ligament. The superficial peroneal nerve is divided proximal to the flap and buried in the muscles. The pedicle is exposed. The posterior margin of the flap is then elevated. At this stage, the flap remains attached only to the septum between the anterior and lateral compartments. The perforating branch of the peroneal artery is then clamped temporarily to see the adequacy of retrograde flow in the skin island **(Fig A)**. If the flow is good, then the perforator is ligated proximal to the emergence of skin perforators. If the flow is deemed unsatisfactory, the flap is sutured back on the bed and delayed for 48 h. In many cases, the peroneal perforator is absent and it is replaced by an ascending perforator called inferolateral collateral artery, a branch of the anterior lateral malleolar artery as shown in **(Fig B)**. This ascending branch inferolateral collateral artery supplies the skin of the anterolateral aspect of the leg up to mid-level. A pre-operative picture of such a case is shown in **(Fig C)**. In the end, the septum is incised sub-periosteally and the flap rotated as a distally based pedicle island to the required defect. Release of the pedicle up to the sinus tarsi enhances rotation by adding length to the pedicle. Division of the fascia on the posterior border of the extensor digitorum brevis helps to avoid compression on the pedicle. The closure of the donor site is achieved by suturing the peroneal and extensor muscles together. A split-thickness skin graft is usually required for coverage of the donor site defect. In the case of peninsular rotation flap, the pedicle may not need exposure, and the flap rotated on a distal hinge. Similarly, for coverage of very distal defects on the bases of toes, a flap based on a compound pedicle can be harvested. In the case of an absent peroneal perforator, the flap may be based on antegrade circulation from the inferolateral collateral artery, a branch of the anterior tibial artery.

**Figure A:**
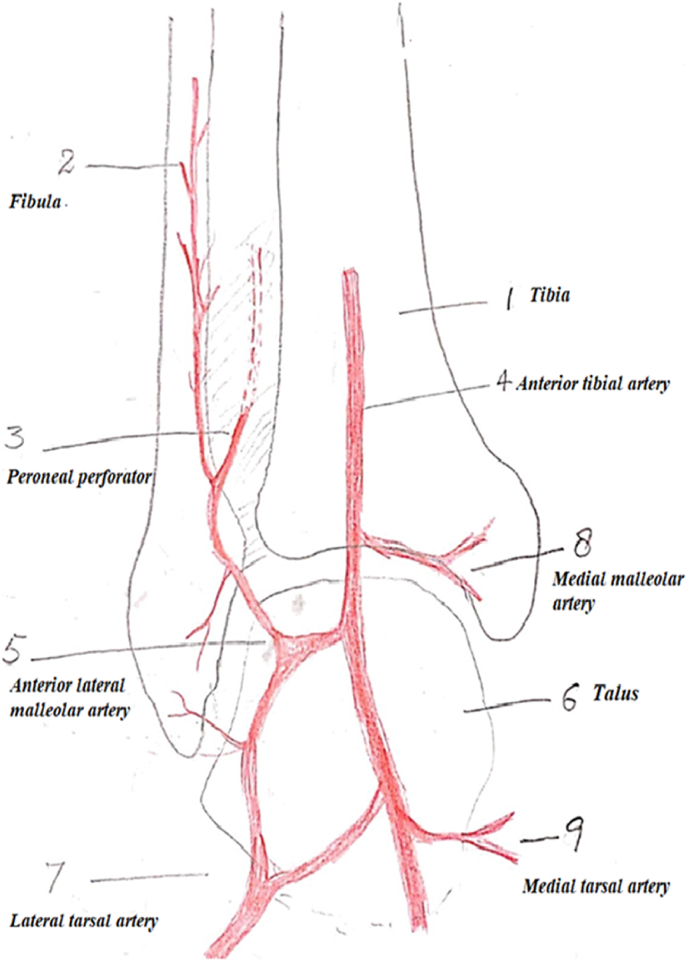
Showing schematic diagram of anastomosis of vessels around ankle and presence of Peroneal perforator.

**Figure B:**
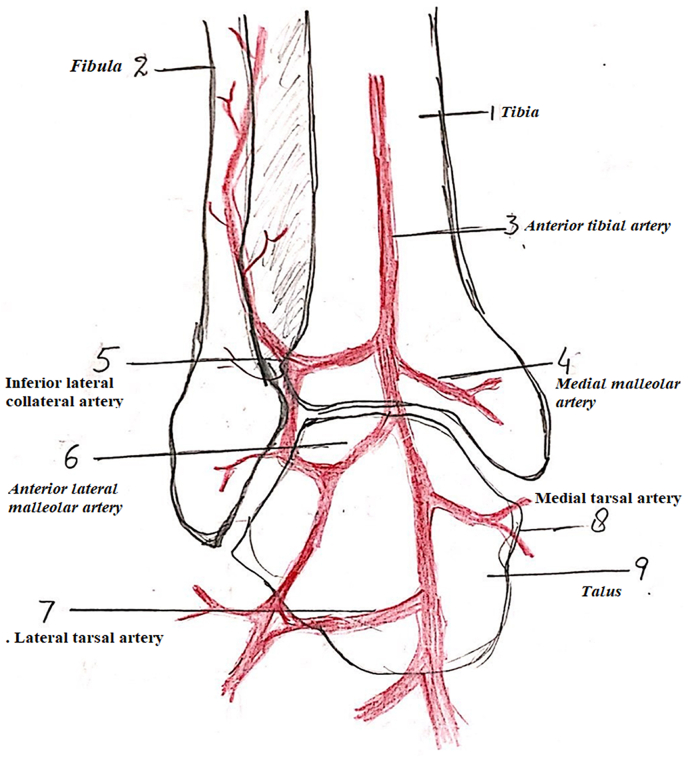
Showing schematic diagram of anastomosis of vessels around ankle and absence of Peroneal perforator and its replacement with inferolateral collateral artery.

**Figure C:**
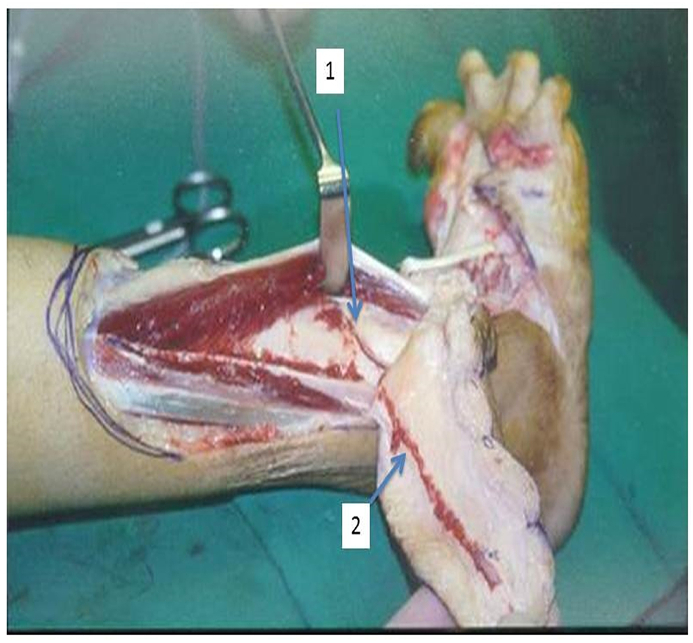
Showing (1) Anterolateral malleolar artrey. (2) Inferolateral malleolar artrey.

### 3.1 Distally based sural artery flap

After the administration of anesthesia (general or spinal) depending upon the patient's condition and the American Society of Anesthesiologists (ASA) classification level, the patient is positioned in supine or lateral decubitus position with the involved side up. The supine position has opted if a concomitant procedure is planned on the foot and ankle; in this situation, a sandbag is placed underneath the buttock. A high-up tourniquet is applied, flap marking is performed, and the level of perforators is marked along the fibula almost 1–1.5 cm posterior to the fibular border **(Figure D).** The neurovascular axis of the flap is marked looking at the surface anatomy of the short saphenous vein; roughly, the axis starts from the midpoint between the lateral malleolus and the attachment of the Achilles tendon (TA) to the middle of the calf in between the two heads of gastrocnemius in the upper one-third of the leg posteriorly. The pivot point of the sural neurovascular island flap is around the last perforator, audible with ultrasound. The last peroneal artery perforator is almost 5–7 cm above the tip of the lateral malleolus. After anesthesia, preparation and draping are performed with a sterile technique. The procedure is performed under a tourniquet with a pressure of 300–350 mgH. Appropriate antibiotics are administered intravenously 20–25 min before inflating the tourniquet. First, the defect site is debrided if required and the dimension of the defect size is measured and documented. The flap outline is marked around the axis of the sural neurovascular pedicle. Then the gauze piece is placed around the marked linings of the outlined flap; this gauze piece is cut around the marking of the flap and rotated at the pivot point of the last perforator. The flap size is kept 2–3 cm c longer in length and 1–1.5 cm wider in dimension. The first incision is made along the posterior margin of the flap. After cutting the skin and subcutaneous tissue skin margins are undermined away from the flap almost a couple of centimeters. Then the deep fascia is cut, and the flap is dissected up and down the posterior incision. **(Figure E)**. As the flap is raised deep to the fascia towards the fibula, the short saphenous vein and sural nerve are identified with their small feeding vessels and lifted with the flap towards the fibula. Close to the fibula, various perforators are identified and dissected carefully till the last major perforator is encountered around the pivot point of the flap rotation, which has to be saved and protected. After this, an anterior incision of the flap is made, skin and subcutaneous tissue are undermined away from the flap; the deep fascia is cut and that deep fascia is incorporated in the flap. The flap is raised with the deep fascia with blunt dissection towards the fibula. During this dissection, one must be careful not to cut the perforators already identified. In the meantime, the saphenous vein and sural nerve are identified at the upper limit of the flap and ligated with a suture. After this, the upper perforators are ligated, except for the last one of the pivot points of rotation that acts as the main source of the retrograde circulation. Once the flap has been raised, the tourniquet is released and removed to avoid venous congestion. The operating surgeon for this series notes that identifying the short saphenous vein near the pivot point and putting a silk ligature around it but not ligating the vein is an important step; the end of the free suture around the vein is left outside of the wound. Postoperatively, if the flap develops venous congestion due to venous drainage of the short saphenous vein, then this vein can be ligated through its free ends outside the wound. In this way, we do not have to take the patient to the operating room for exploration of the vein and subsequent ligation [33]. Once the flap has been rotated to the recipient site and the flap is sutured to the skin of the recipient area, the donor site is closed primarily or with a skin graft taken from the same thigh. **(Figure F)**.

**Figure D:**
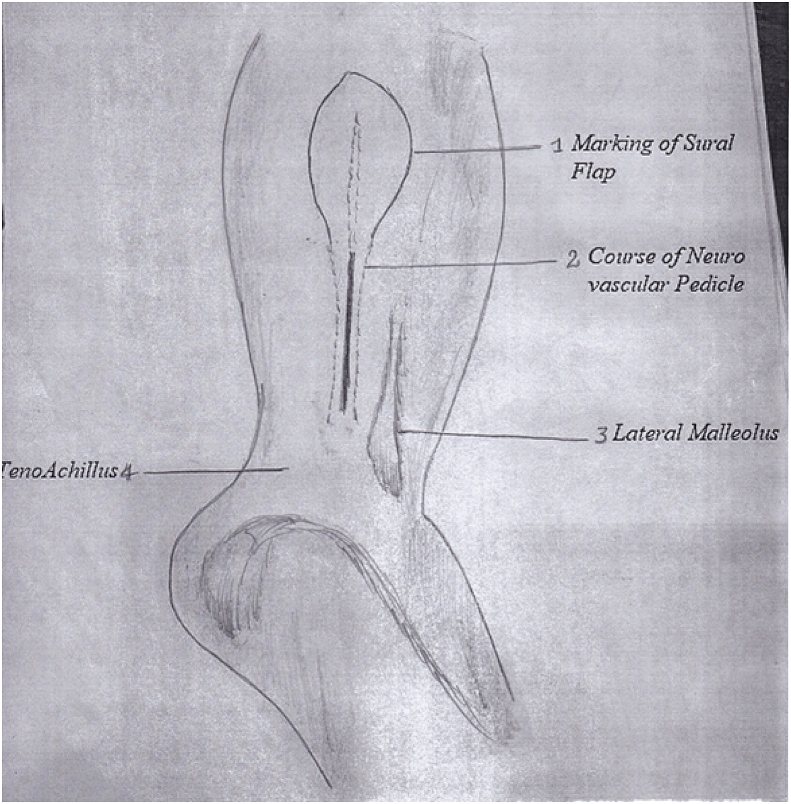
Steps of flap dissection.

**Figure E:**
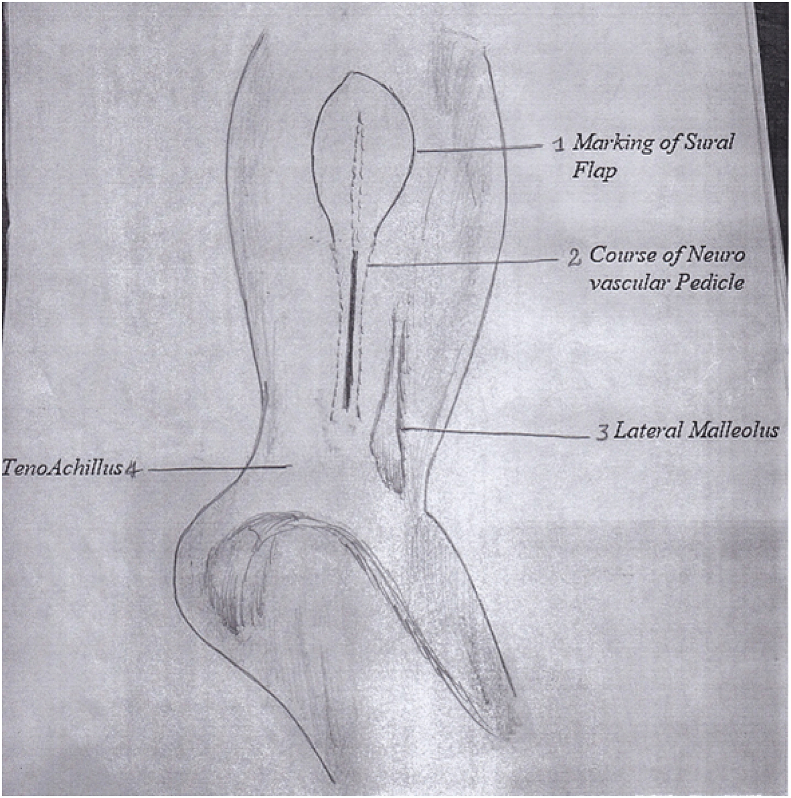
Dissection of neurovascular.

**Figure F:**
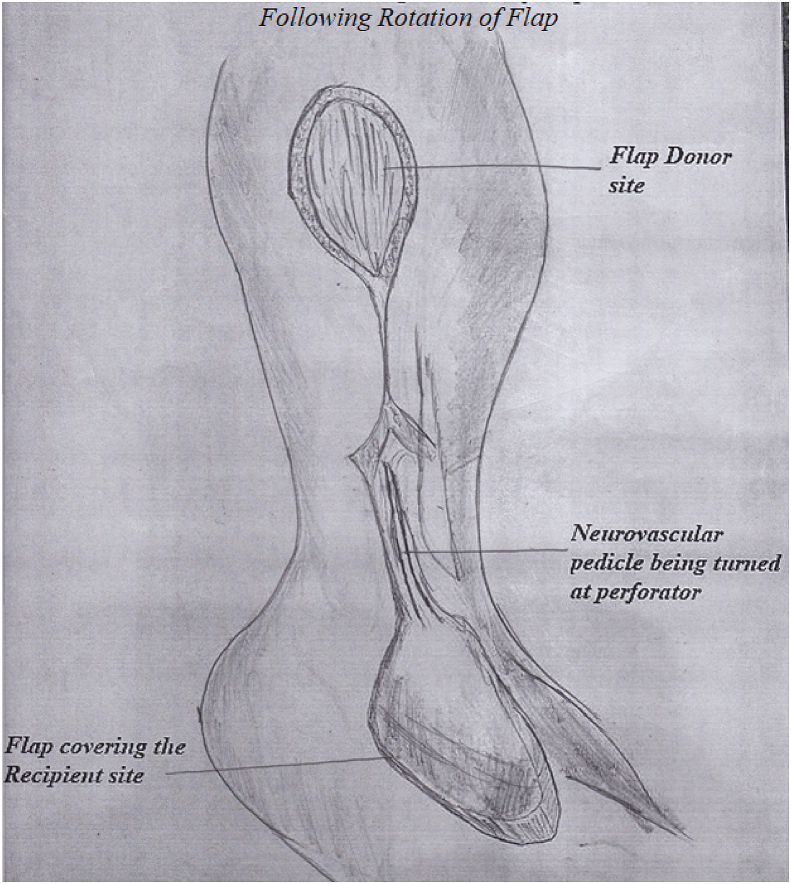
Following rotation of flap.

## 4 Result

Over 25 years from 1996 to 2020, a retrospective analysis of 53 patients with 27 (51%) cases of sural artery flap and 26 (49%) cases of Supramalleolar Flap were performed for the reconstruction of soft tissues defect of the dorsum of foot, ankle, and perimalleolar area. The male to female ratio was 2.3:1. The mean ages at the time of operation were 26.76 ± 22 years (range, 4 months to 74 years) for the supra-malleolar flap and 32.70 ± 19.62 years (range, 2–69 years) for the sural artery flap. The mean follows up of 12 years for sural artery flap and 6 years for supramalleolar (range; 2–268 months). The right to left side ratio was 1.3:1 in both flaps in which the most common cause of the defect was trauma (32 cases; 60.4%). The maximum flap size harvested was 25*10 cm for the sural artery flap and 20*8 cm for the supramalleolar flap. The clinical details of patients were divided into two groups for long-term clinical and functional outcomes of the flap; group I included the sural artery flap and group II included the Supramalleolar flap. (Table 1). Final follow-up of all patients was done in the clinic and the functional status for both flaps was assessed by Self designed tool and was named as Hashmi Flap Outcome Score (HFOS) (Table 2).

**Table 1:** Clinical Summaries of 53 patients with two different flaps.

S.no	Variables		Flap Types		Significance P-value
			Sural artery Flap (N = 27 cases)	Supramalleolar Flap (N = 26 cases)	
1.	Gender	Male	18 (66.7%)	19 (73.1%)	0.619
		Female	9 (33.3%)	7 (26.9%)	
2.	Age	Mean ± SD (in years)	32.70 ± 19.72	26.7 ± 21.8	0.301
3.	Mechanism of Injury	Trauma	17 (63%)	15 (57.7%)	0.476
		Infection	2 (7.4%)	7 (27%)	
		Blast injury	3 (11.1%)	1 (3.8%)	
		Contracture release	3 (11.1%)	2 (7.7%)	
		TA coverage	2 (7.4%)	0	
		Tumor	0	1 (3.8%)	
4.	Defect Side	Right	16 (59.3%)	14 (53.8%)	0.698
		Left	11 (40.7%)	12 (46.2%)	
5.	Flap Size	Mean in cm (L + B)/2	12.35 ± 3.48	9.12 ± 1.53	0.000
6.	Complications	Partial necrosis	1 (3.7%)	2 (7.7%)	0.450
		Infections	2 (7.4%)	0	
		Venous Congestions	1 (3.7%)	2 (7.7%)	
		No Complications	23 (85.2%)	22 (84.6%)	
7.	Rate of Flap Survival (based on Flap coverage)		96.3%	96.2%	0.672

**Table 2:** Flap outcome grade by self-designed tool.

Variables	Excellent (5)	Good (4)	Fair (3)	Poor (2)
**Coverage**	100%	90–100%	80–90%	50–70%
**Cosmetic Appearance**	Highly acceptable	Acceptable with slightly raised skin margins	Acceptable with raised skin margins	Not acceptable due to thick and hairy skin
**ADL**	No issue in ADL and sports	No issue in ADL, difficulty in sports	The mild issue in ADL, cannot play sports	Difficulty in ADL and sports
**Weight-bearing**	Full weight-bearing	Full weight-bearing mild discomfort in sport	Discomfort in full weight-bearing	Pain on full weight-bearing
**Total Score**	20	16	12	8

**Lateral Supramalleolar Flap:** Twenty-two out of 26 flaps (84.6%) had complete healing without complication while 4 (15.4%) patients had complications; two had partial necrosis and two had venous congestion. 25 patients had 100% coverage of flap and were classified as Excellent. Two patients with partial necrosis did not require skin graft as necrosed skin was re-epithelized with daily dressing. Cosmetically, it was graded as Excellent in 22 patients and 4 patients complained of thick flap requiring de-fattening. The donor site was cosmetically acceptable to the majority of the patient. 23 (88.5%) patients had full weight-bearing and excellent activities of daily living. 3/4 of patients requiring defatting had improved in weight-bearing status and had no issue with shoe wearing while one patient had problems with shoe wear following defatting. The final grading can be seen in Table 3.

**Table 3:** Comparative Outcomes of Sural artery flap and Supramalleolar flap (based on Table 2).

Outcome Scores	Group I (Sural artery flap) N = 23	Group II (Supramalleolar flap) N = 24	Significance P values
Excellent	19 (70.4%)	24 (92.3%)	0.033
Good	6 (22.2%)	2 (7.7%)	
Fair	2 (7.4%)	0	
Poor	0	0	
Mean score ± S. D	17.78 ± 2.87	19.19 ± 1.4	0.028

**Distally based Sural artery flap:** Twenty-three out of 27 flaps (85.2%) showed complete healing with no complication while 4 patients had flap complications; two had infections requiring debridement. One had partial necrosis and one had venous congestion. 26 patients had 100% coverage of defect site and graded as excellent while Only 1 patient had partial coverage of defect (80%) requiring skin grafting. The cosmetic appearance was acceptable to 19 patients graded as excellent while 6 patients complained of the thick flap with raised skin margins (graded as good) and two patients mentioned it thick and hairy graded as Fair. 18 (66.7%) had full weight-bearing (FWB) and no issue in activities of daily living (ADL). Nine patients had mild discomfort in FWB and had no issue in ADL graded as Fair as mentioned in Table 3.

### 4.1 Comparison of both flap types

There was not any significant difference between the two flaps in terms of age, gender, cause of the defect, defect side, complications, and rate of flap survival. However, we observed a significant difference in the HFOS between the two groups at a 95% confidence interval. (*p* = 0.028). (Table 3).

## 5 Discussion

The management of soft tissue defects over the foot and ankle has always been challenging for plastic, reconstructive and orthopedic surgeons. Because of thin skin coverage, this region is more exposed to trauma and requires good durable skin coverage. The replacement with thin, sensate, and the same kind of skin is almost impossible. Most of the reconstructive flap procedures provide thick and hairy skin to this region that may cause a problem in show wear, unacceptable cosmetic appearance, and weight-bearing issues in daily living activities. The feet carry the weight of the body while standing, walking and to achieve this purpose restoration of the sensation of the foot is the key factor for a stable reconstruction [22]. Several reconstructive procedures like local neurovascular island flaps and free flaps have been described on the distal lower extremity [23,24]. The comparisons between different flaps on the same site have been reported in the literature during the past two decades [[25], [26], [27], [28]]. Each flap has its characters, advantages, and disadvantages. Zhu YL [25] compared 62 pedicle flaps with 164 free flaps for the foot and ankle reconstruction and found the pedicle flaps were the best option with a 4.4% complication rate than free flaps. Similarly, a recent study 2019 [26] demonstrated the additional advantages of fasciocutaneous flaps for the defects of the foot and ankle region with fewer rates of complications with muscle flaps (7.3% vs. 19%). These studies reported the comparison between free flaps and local fasciocutaneous flaps and preferred local regional fasciocutaneous flaps over free flap groups.

Touam C et al. [29] compared the outcome of 42 cases of sural artery flaps with 27 Supramalleolar flaps for coverage of lower leg, ankle, and foot and perimalleolar areas. They had complete necrosis of 3 cases of Supramalleolar and 2 cases of sural artery flaps. They concluded that the sural artery flap is better in terms of survival as they had necrosis of 3 cases of Supramalleolar flaps.

Xu Gong et al. [30] compared the sural artery flap with Supramalleolar flaps. They had compared 15 cases of Supramalleolar with 22 cases of sural artery flap. They had complete necrosis in 3 and partial necrosis out of 15 Supramalleolar flaps. On the contrary, they had only one case of complete necrosis in sural artery flaps out of 22. They concluded that lateral Supramalleolar flaps are more suitable for the skin defect of a smaller area over the medial or lateral malleolus and proximal dorsum of the foot. According to this series, the reverse sural neurocutaneous flaps are more suitable for the skin defect of a larger area over the foot and ankle without serious destruction of the malleolar arterial rete.

The above two studies had done a comparison of these two flaps for coverage of defects around the lower leg, ankle, perimalleolar areas, and dorsum of the foot. But none of the studies have done a comparison of these two flaps for reconstruction of extensive defects of the dorsum of the foot and ankle. Our study has focused only on coverage and reconstruction of the dorsum of the foot and ankle only. We have not included those cases of both flaps done for coverage of the lower leg, heel, and perimalleolar area. This is a big difference between the previous studies and mine. There is tremendous literature support in favor of sural artery flap for coverage of lower leg, foot, ankle, and perimalleolar areas [20,23,[29], [30], [31], [32]].

Twenty-Six cases of Supramalleolar and 27 cases of Sural artery flaps were treated for coverage of dorsum of foot and ankle; the flaps did for perimalleolar area, heel, and lower legs were not included in the comparison. Our study found significantly lower rates of wound complication (15%) in both flaps, partial tip necrosis in 2 cases of Supramalleolar and one case of sural artery flap while two cases had a deep infection requiring debridement and three cases had venous congestion.

Based on the anatomy, its vascular territory, the pivot point of rotation, ease of dissection, and pros and cons of both flaps; we noted the following difference in both flaps as highlighted in Table 4.

**Table 4:** Comparative Analysis of both flaps based on anatomy.

Sr. No	TYPE OF FLAP	SURAL ARTERY FLAP	SUPRAMALLEOLAR FLAP
1.	Anatomical basis of flap vascularity	Segmental sural artery accompanying sural Nerve and small saphenous vein	Terminal perforator of peroneal artery after piercing the interosseous membrane or anterolateral malleolar artery
2.	Dimension of flap	10–25 cm in length and 7–10 cm in width	10–20 cm in length and 7–9 cm in width
3.	Pivot point of rotation	6–9 cm above the tip of lateral malleoleus	Around the anterior aspect of ankle joint or distal to ankle joint at sinus tarsi
4.	Scarification of neurovascular structures	It sacrifices the sural nerve and small saphenous vein	Does not sacrifice any major vessel or nerve
5.	Position of patient	Procedure can be done in supine but one has to change the position of leg. Better to perform procedure in lateral position	Procedure is done in supine position only
6.	Advantages	Very large area of skin can be harvested	Harvested skin area is relatively small as compared to sural artery flap
7.	Disadvantages	a) Scarification of saphenous vein and sural nerve	a) No scarification of major vein, artery or nerve
		b) Very large flap has to be harvested if dorsum of foot is reconstructed with sural flap as pivot point of rotation is 7 cm above the ankle joint	b) Harvested flap size is almost equivalent to defect size.
		c) High chances of venous congestion due to drainage of venous blood in the intact saphenous vein	c) No excessive venous congestion.
		d) Thick and hairy	d) Thick and hairy

The important point is getting the extra length of the sural artery flap for coverage of the dorsum of foot and ankle as the pivot point of rotation is 5–7 cm above the tip of lateral malleolus while the pivot point of rotation for Supramalleolar flap is at the ankle joint. Keeping this factor in mind we need a shorter length of pedicle for the Supramalleolar flap as compared to the sural artery where we need the extra length of pedicle and flap both for coverage of dorsal foot defects.

The most important feature of this study is an objective clinical and functional analysis of flaps based on coverage of defects, cosmetic appearance, weight-bearing status, and ADL which was done by a Self-Designed Scoring Tool. This tool is validated [33] and has been used to measure the outcomes of flaps around the lower extremity. Based on that tool, the majority of our cases of supramalleolar flap fall in the excellent category (92.3%) as compare to Sural (70.4%). The percentage of good category for Supramalleolar flap was 7.7% and 22.2% for sural artery flaps as these cases required coverage of defects around foot and ankle affecting the functional outcome.

Our experience differed from that of Touam et al. [29] who found a greater number of complications when lateral supramalleolar flaps were chosen and testified that the distally based sural artery flap is more reliable especially regarding the venous congestion. Several authors concentrated mainly on the use of a sural flap [13,15,16]. The disadvantages are thick, hairy, and insensate and need good knowledge of anatomy and proper dissection. It causes an ugly scar on the back of the calf which is especially not acceptable to women and leads to permanent loss of sensation in the distribution of the sural nerve on the lateral border of the foot and 5th toe. The donor site usually requires skin grafting, occasionally it can be closed in case of smaller flaps and older age groups when skin is lax. The skin closure is also made easier if a flap is raised in an elliptical fashion, which decreases the chances of skin grafting. Another innovation we did in surgical technique that we identify the short saphenous vein near the pivot point and putting a silk ligature around it but do not ligate the vein; an important step. The ends of the free suture around the vein are left outside of the wound. Postoperatively, if the flap develops venous congestion due to venous drainage of the short saphenous vein, then this vein can be ligated through its free ends outside the wound. In this way, we do not have to take the patient to the operating room for exploration of the vein and subsequent ligation. The flap vascularity can be increased when both neurocutaneous and veno cutaneous vessel (sural nerve and saphenous vein) both are incorporated in the pedicle of the flap.

Similarly, we adapted two measures in the case of the Supramalleolar flap to increase its vascularity and decrease venous congestion. The operating surgeon always dissects the terminal branch of the peroneal artery that pierces the interosseous membrane 3–4 cm proximal to the ankle joint and gives a cutaneous branch to the skin in T shaped or Y shaped manner; the ascending branch supplies the skin of the anterolateral aspect of the leg and descending branch anastomosis with ascending branch of the anterolateral malleolar artery as seen in **(Fig A)**. In case the terminal branch of the peroneal artery is absent or rudimentary; it is replaced by the anterolateral malleolar artery. **(Fig B**). Based on this dissection, it is decided per-operatively that flap will be retrograde or antegrade. In the case of retrograde, the pedicle length can be long and dissected up to sinus tarsi but in the case of antegrade, the pedicle length will be short as the flap is based on the anterolateral malleolar artery. The second modification we did was delayed in rotation of Supramalleolar for 2–3 days till the choke vessels of flap open and circulation improve and the flap is rotated to the recipient site after 2–3 days.

Our results do not match with the findings of other authors**.** [20,[29], [30], [31], [32]] Analyzing our results and comparison between both flaps, we found that lateral supramalleolar artery flap provides excellent coverage to the dorsum of the foot up to the metatarsophalangeal joint. The advantages of the flap are easy dissection, robust pliable skin, flexibility in rotation of flap, and allow excellent coverage of dorsal skin defects of ankle and feet up to toes contrary to the belief that it cannot cover the dorsum of foot [31]. On the other hand, the Sural artery flap is an excellent flap to cover the perimalleolar area, ankle, and dorsal aspect of the foot. But we have to do extra dissection and harvest a bigger flap as the pivot point of rotation is 5–7 cm above the tip of the lateral malleolus as we need to preserve the last perforator of the peroneal artery to have an intact circulation of the flap.

Choosing the flaps to treat the wounds of this region must follow the basic requirements: the flap should be large enough to cover the defect, less bulky to allow for reasonable contour, should contain sensory nerves for protective sensation [32] Unfortunately both these flaps are insensate flaps and relatively thick and hairy. Many patients do ask for hair follicle ablation and defatting as a sequel.

## 6 Conclusion

In conclusion, both sural artery flap and lateral supramalleolar flaps have proven reliable, versatile, and effective choice of reconstruction for medium to large size wounds, even in debilitated patients. The lateral Supramalleolar flap is the first choice for reconstruction of dorsal defect of the ankle and foot provided the surgeon is experienced and dissects the pedicle flap first to determine the mode flap elevation retrograde or antegrade. Based on the vascular axis, it has a large skin paddle and a wide rotation arc that reaches the distal areas of the foot and allows excellent coverage with low complication and facilitates good outcomes. The sural artery flap can cover larger defects as compared to the Supramalleolar flap but the arc of rotation is around the last peroneal perforator that is 5–7 cm above the tip of the lateral malleolus.

## Financial disclosure statement

The principal author has nothing to declare. No financial benefit or support was taken from any source.

## Data statement

Data will be made available on request.

## Ethical approval

Approved by ERC committee and ERC number is 4452.

## Sources of funding

We have no affiliation with or financial involvement with any organization or entity with financial or other interest in the matter discussed in the manuscript.

Dr Pervaiz Mehmood Hashmi: Study design and manuscript writing.

Abeer Musaddiq: Study approval, data collection, study, manuscript writing, data analysis.

Alizah Hashmi: Contributors.

Dr Marij Zahid: Contributors.

## Registration of study

Name of the registry: ClinicalTrials.gov PRS (Protocol Registration and Results System).

Unique Identifying number or registration ID: NCT05027542.

Hyperlink to your specific registration (must be publicly accessible and will be checked): https://clinicaltrials.gov/ct2/show/NCT05027542.

## Guarantor

Dr Pervaiz Hashmi is the one who accepts the official responsibility of this research study that includes Ethics (approved by ERC), data handling, reporting of results and study conduct.

## Provenance and peer review

Not commissioned, externally peer-reviewed.

## Declaration of competing interest

None.

## Figures and Tables

**Case 1:**
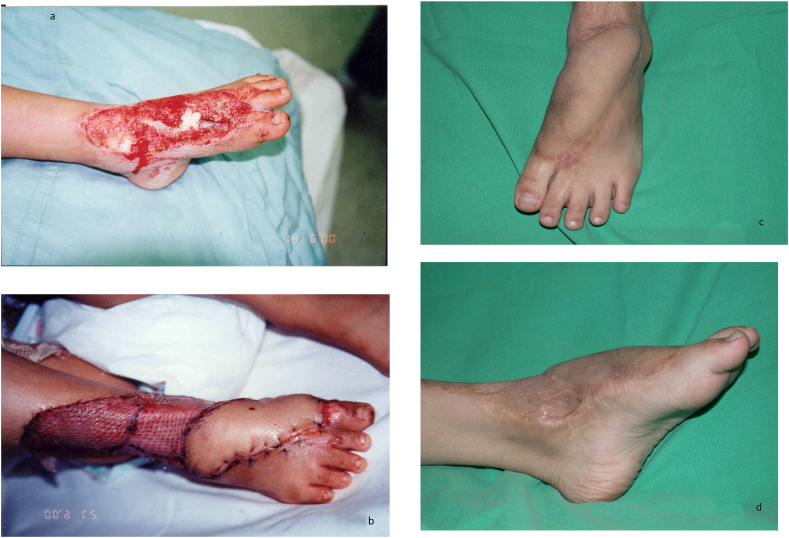
**RECONSTRUCTION OF DORSUM OF FOOT AND ANKLE WITH SUPRAMALLEOLAR FLAP:** This 4 years girl sustained a grade III-B fracture of the first metatarsal of the left foot as a result of a runover injury. The area of degloving extended from the dorsum of the foot and ankle up to the bases of the first and second toes (Figure 1. a). To cover this defect, a lateral supramalleolar flap was raised as a retrograde pedicle island flap (Fig. 1 b). Three and a half years postoperatively after de-fattening of the flap for cosmetic reasons (Figure 1. c and 1.d), an excellent range of ankle motion was achieved.

**Case 2:**
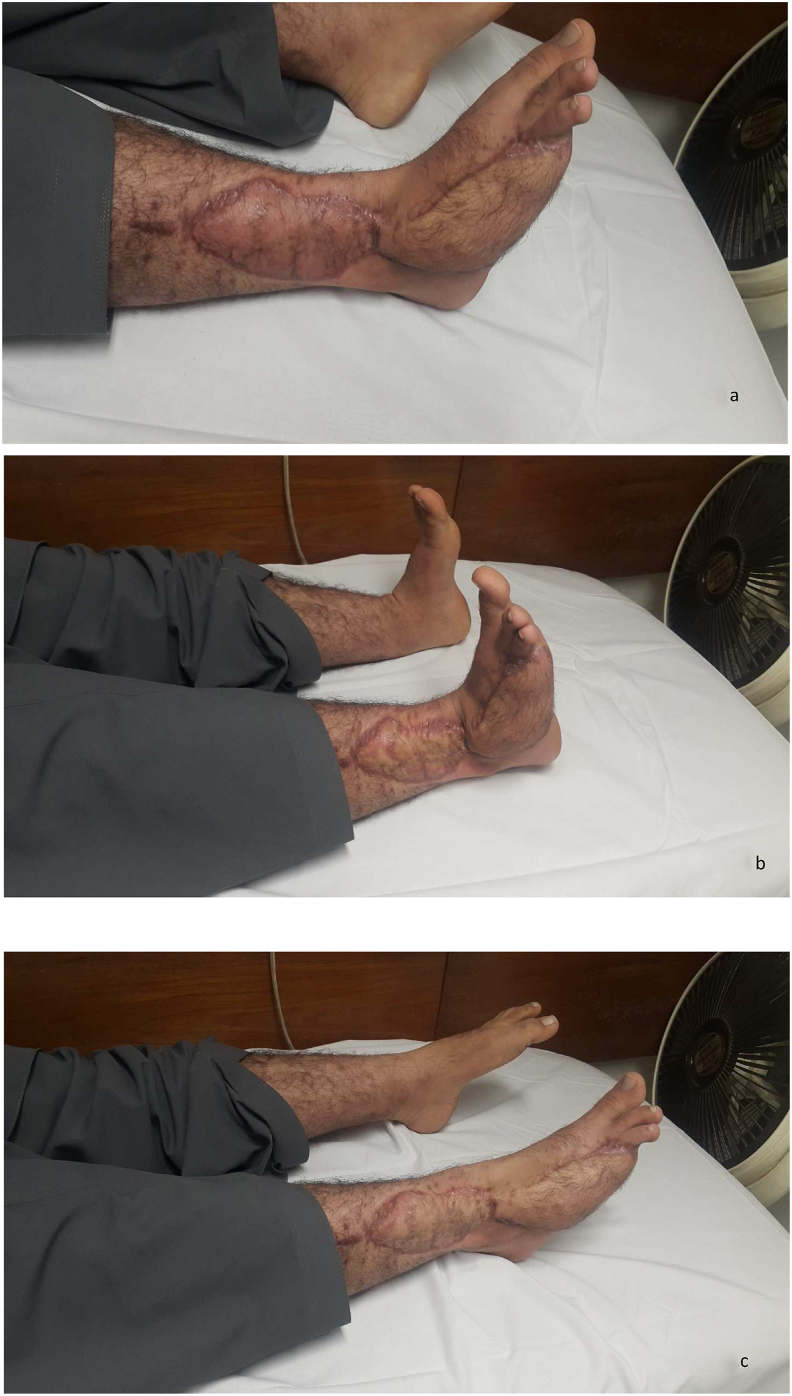
**COVERAGE OF DORSAL FOOT DEFECT WITH SUPRAMALLEOLAR FLAP** 33 tears old gentleman had a road traffic accident when a wheel of a vehicle ran over his foot and lead to degloving injury of his foot with amputation of 4th and 5th toes. The defect was covered with a supramalleolar flap. Fig. 2. shows postoperative coverage of defect. Fig. 2.b shows full dorsiflexion and Fig. 2.c shows plantar flexion.

**Case 3:**
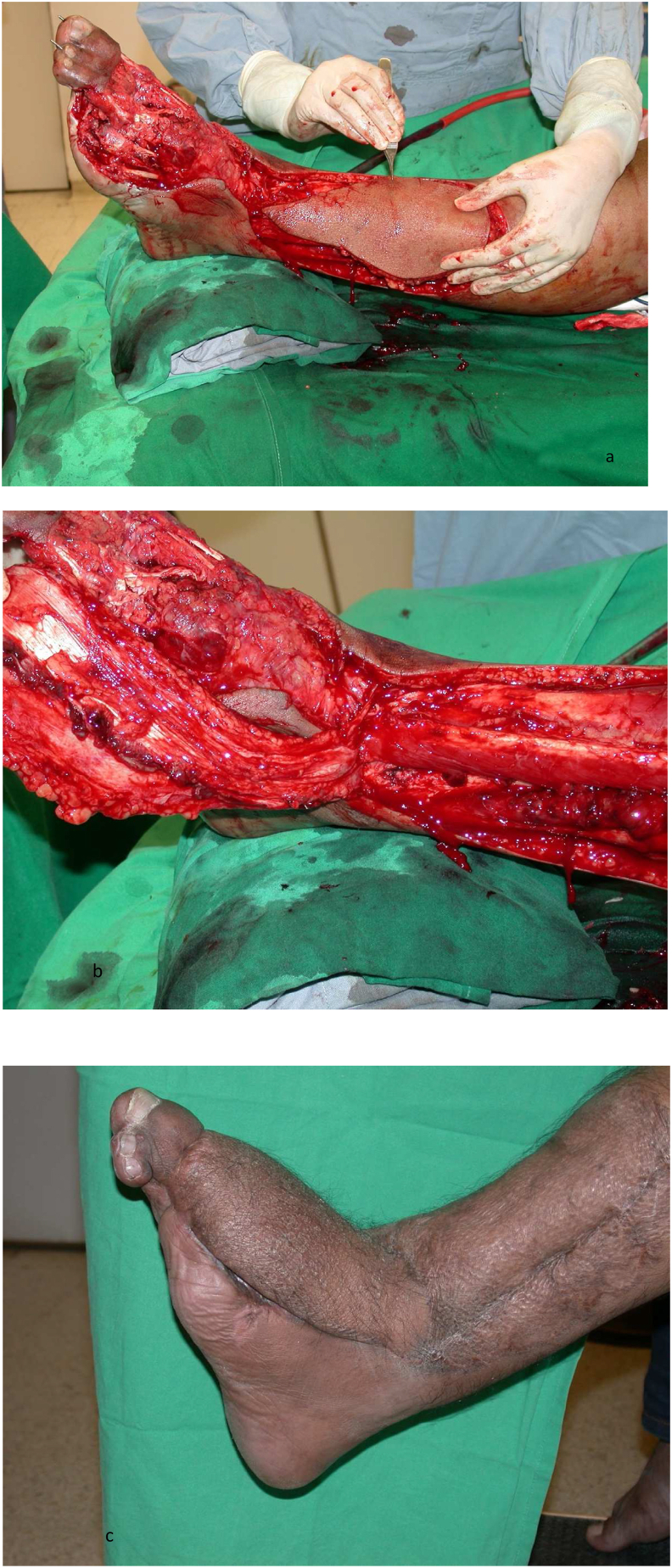
COVERAGE OF DORSAL FOOT DEFECT WITH SUPRAMALLEOLAR FLAP 38 years old gentleman sustained an injury to his left foot in a road traffic accident when the tire of a vehicle ran over his foot. There was the loss of lateral two toes and skin over the dorsum of the foot. Following debridement, the defect was covered with a supra malleolar flap, based in an inferior lateral collateral artery (Fig. 3.a and 3.b). Excellent outcome after three years (Fig. 3.c)

**Case 4:**
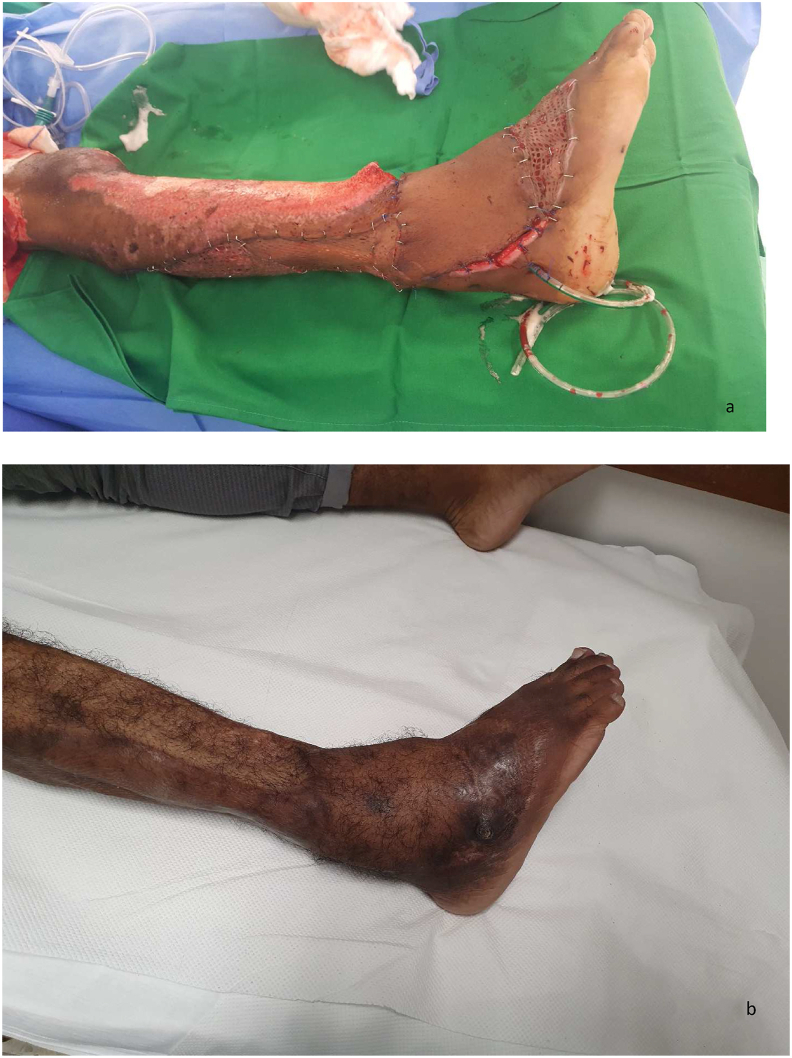
**RECONSTRUCTION OF DORSAL SKIN DEFECT WITH SURAL ARTERY FLAP** A young man had a vehicle run-over injury on his right leg that resulted in the loss of dorsal skin around the distal leg, ankle joint, and foot. This was reconstructed with a sural artery flap. Fig. 4.a shows immediate per-operative picture, Fig. 4.b shows postoperative results after 9 months of wound coverage.

**Case 5:**
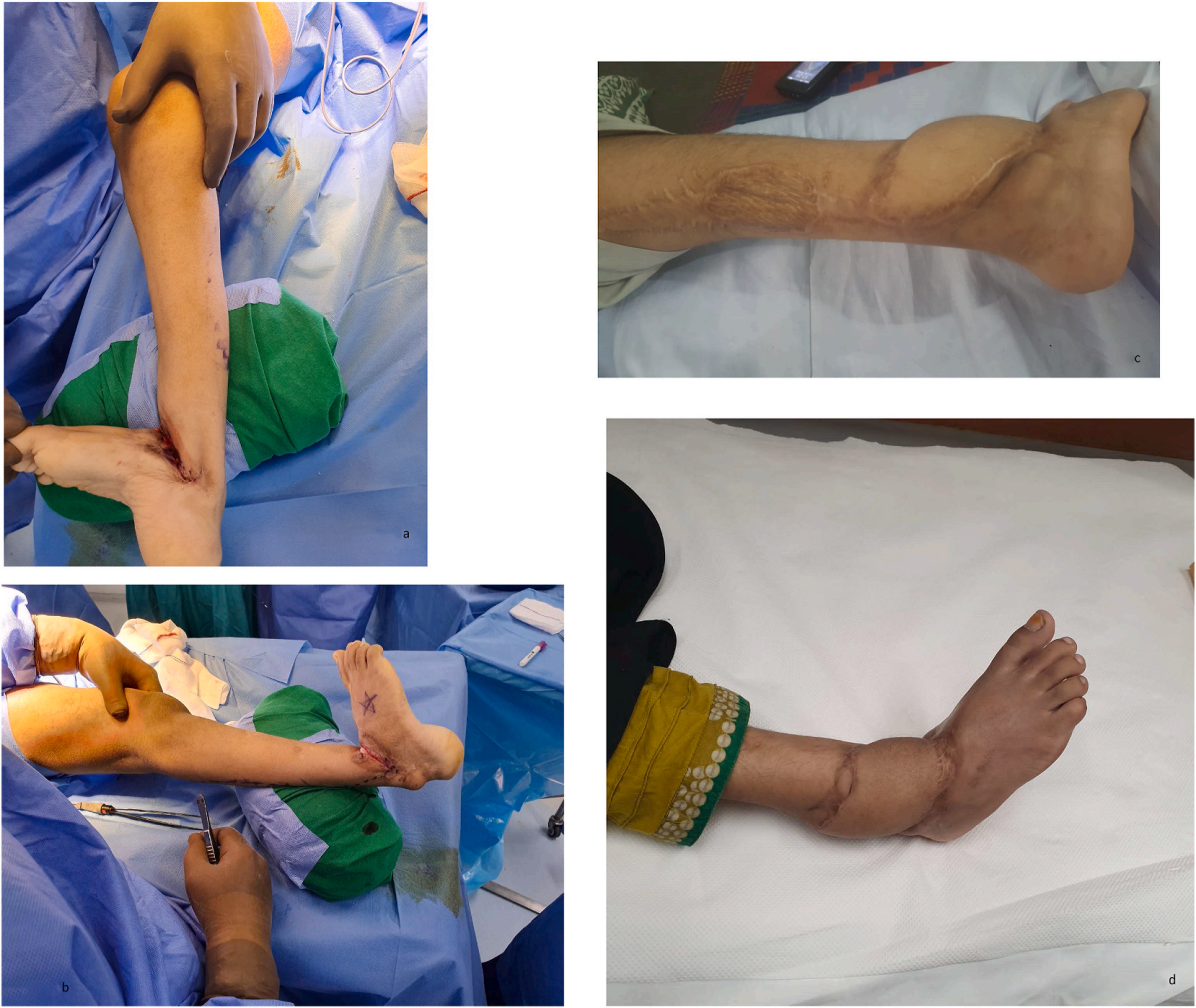
COVERAGE OF DORSUM OF FOOT WITH SURAL ARTERY FLAP 17 years old girl presented with post-traumatic contracture and calcaneal foot deformity. Fig. 5.a and 5.b shows contracture was released and reconstructed with a sural artery flap. Fig. 5.c shows the postoperative result after 12 months. Fig. 5.d shows an Excellent outcome after 8 years.

**Case 6:**
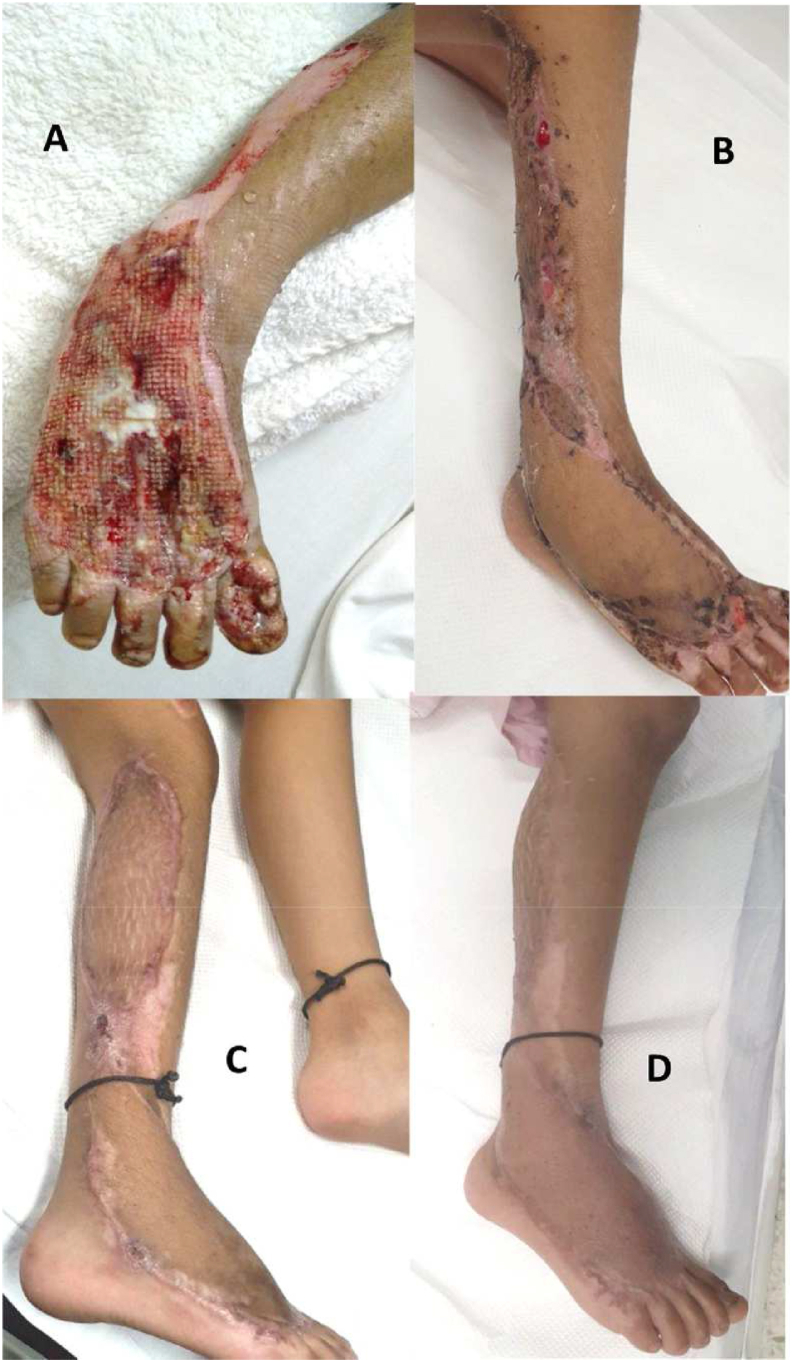
**COVERAGE OF DORSAL SKIN DEFECT WITH SURAL ARTERY FLAP** 9 years old girl with a history of Road Traffic Accident, had extensive soft tissue injury over the dorsum of the foot and lateral aspect of the ankle. A: shows wound after multiple debridements; Fig B illustrates 3 weeks post flap coverage. Figures C and D show the postoperative result after 6 months
